# Does peer-navigated linkage to care work? A cross-sectional study of active linkage to care within an integrated non-communicable disease-HIV testing centre for adults in Soweto, South Africa

**DOI:** 10.1371/journal.pone.0241014

**Published:** 2020-10-22

**Authors:** Kathryn L. Hopkins, Khuthadzo E. Hlongwane, Kennedy Otwombe, Janan Dietrich, Maya Jaffer, Mireille Cheyip, Jacobus Olivier, Heidi van Rooyen, Alisha N. Wade, Tanya Doherty, Glenda E. Gray

**Affiliations:** 1 Perinatal HIV Research Unit, Faculty of Health Sciences, University of the Witwatersrand, Chris Hani Baragwanath Academic Hospital, Johannesburg, South Africa; 2 School of Public Health, Faculty of Health Sciences, University of the Witwatersrand, Johannesburg, South Africa; 3 Health Systems Research Unit, South African Medical Research Council, Cape Town, South Africa; 4 Centers for Disease Control and Prevention, Pretoria, South Africa; 5 Human and Social Development Programme, Human Sciences Research Council, Pretoria, South Africa; 6 School of Clinical Medicine, Faculty of Health Sciences, University of the Witwatersrand, Johannesburg, South Africa; 7 MRC/Wits Rural Public Health and Health Transitions Research Unit, School of Public Health, Faculty of Health Sciences, University of the Witwatersrand, Johannesburg, South Africa; 8 School of Public Health, University of the Western Cape, Cape Town, South Africa; 9 Office of the President, South African Medical Research Council, Cape Town, South Africa; Universiteit Maastricht, NETHERLANDS

## Abstract

**Introduction:**

South Africa is the HIV epidemic epicentre; however, non-communicable diseases (NCDs) will be the most common cause of death by 2030. To improve identification and initiation of care for HIV and NCDs, we assessed proportion of clients referred and linked to care (LTC) for abnormal/positive screening results and time to LTC and treatment initiation from a HIV Testing Services (HTS) Centre before and after integrated testing for NCDs with optional peer-navigated linkage to care.

**Materials and methods:**

This two-phase prospective study was conducted at an adult HTS Centre in Soweto, South Africa. Phase 1 (February-June 2018) utilised standard of care (SOC) HTS services (blood pressure [BP], HIV rapid diagnostic testing (RDT), sexually transmitted infections [STI]/Tuberculosis [TB] symptom screening) with passive referral for abnormal/positive results. Phase 2 (June 2018-March 2019) further integrated blood glucose/cholesterol/chlamydia RDT, with optional peer-navigated referral. Enrolled referred clients completed telephonic follow-up surveys confirming LTC/treatment initiation ≤3 months post-screening. Socio-demographics, screening results, time to LTC/treatment initiation, peer-navigated referral uptake were reported. Analysis included Fisher’s exact, chi-squared, Kruskal Wallis, and Student’s T-tests. Thematic analysis was conducted for open-ended survey responses.

**Results:**

Of all 320 referrals, 40.0% were HIV-infections, 11.9% STIs, 6.6% TB, and 28.8% high/low BP. Of Phase 2-only referrals, 29.4% were for glucose and 23.5% cholesterol. Integrated NCD-HTS had significantly more clients LTC for HIV (76.7%[n = 66/86] vs 52.4%[n = 22/42], p = 0.0052) and within a shorter average time (6–8 days [Interquartile range (IQR):1–18.5] vs 8–13 days [IQR:2–32]) as compared to SOC HTS. Integrated NCD-HTS clients initiated HIV/STIs/BP treatment on average more quickly as compared to SOC HTS (5 days for STIs [IQR:1–21], 8 days for HIV/BP [IQR:5–17 and 2–13, respectively] vs 10 days for STIs [IQR: 4–32], 19.5 days for HIV [IQR:6.5–26.5], 8 days for BP [IQR:2–29)]. Participants chose passive over active referral (89.1% vs 10.9%; p<0.0001). Participants rejecting peer-navigated referral preferred to go alone (55.7% [n = 39/70]). Non-LTC was due to being busy (41.1% [n = 39/95]) and not being ready/refusing treatment (31.6% [n = 30/95]). Normalised results assessed at referral clinic (49.7% [n = 98/196]), prescribed lifestyle modification/monitoring (30.9% [n = 61/196]), and poor clinic flow/congestion and/or further testing required (10.7% [n = 21/196]) were associated with non-treatment initiation.

**Conclusion:**

Same-day treatment initiation is not achieved across diseases, despite peer-navigated referral. There are psychosocial and health systems barriers at entry to care/treatment initiation. Additional research may identify best strategies for rapid treatment initiation.

## Introduction

Since 2015, the Joint United Nations Programme on HIV/AIDS (UNAIDS) has been driving the elimination of the human immunodeficiency virus (HIV) pandemic by 2030 as a threat to global health. UNAIDS’s fast-tracked strategy was to implement the 90-90-90 targets so that by 2020, 90% of all people living with HIV will know their HIV status, 90% of all people with diagnosed HIV infection will receive sustained antiretroviral therapy (ART), and that 90% of all people receiving antiretroviral therapy will have viral suppression [1]. South Africa contains 20% of the global HIV burden [2]. As of 2018, South Africa’s progress in the prevention, treatment and control of HIV fell below the 90-90-90 targets. Encouragingly, 90% of all people living with HIV (PLHIV) within South Africa knew their status, yet only 68% of PLHIV who knew their status are on ART, and of those, 87% were virally supressed. This equated to 61% of all PLHIV in South Africa initiated on sustained ART and 53% of all PLHIV virally suppressed. These data highlight the ongoing challenges within the HIV Care Cascade of severe patient attrition from HIV-diagnosis to treatment initiation.

Furthermore, the disease profile of South Africa is changing rapidly, with the prevalence of chronic non-communicable diseases (NCDs) predicted to become the most common causes of death by 2030 in Africa [3]. There is evidence that HIV-infection, itself, and ART use might be risk factors for NCDs due to causing a persistent inflammatory state and immune dysfunction mechanisms associated with metabolic syndrome diseases [4, 5]. While national and global policies stress the integration of screening and treatment for NCDs within established disease programmes, such as HIV, to address the rising epidemic [6, 7], notably there has yet to be a standard of care NCD protocol rolled out. Evidence from high income countries–where the NCD epidemic originated–shows it is possible to delay NCD-related morbidity and mortality by several decades, avoiding mortality amongst the aging population and producing cost-savings, if screening and treatment are initiated earlier in life [8–10]. We recently reported the feasibility of integrating rapid screening for NCD markers into the standard of care HTS clinic flow, the associated high-level of HTS client acceptability and satisfaction with the services, as well as the high quality of test results stemming from the task-shifting of the integrated rapid testing to lay counsellors [11]. However, it has not yet been determined if the integrated screening progressed to increased linkage to care and treatment [11].

Many of the barriers towards patient linkage to care and retention in the treatment cascade are consistently reported across both chronic communicable and non-communicable disease (NCDs) platforms [12]. Patient healthcare-seeking behaviour may be positively or negatively influenced at multiple levels: individual (e.g., acceptance of result, coping ability), relationship (e.g. social support, risk of intimate partner violence [IPV]) [13], community (e.g. stigma) and health systems (e.g. prior negative experiences with the health system which lead to lack of trust) [14].

In recognizing the anticipated impact of NCDs on both the general population and amongst PLHIV, it becomes critically important to pursue evidence-based strategies that improve linkage to care and treatment [14]. Passive referral (e.g.; only providing names and contact information for care and treatment services) is widely implemented in South Africa’s public health sector across all platforms [15]. Passive referral leaves patients to overcome any psychosocial barriers on their own. Active referral is a more facilitated approach, assisting the patient to remain engaged within the healthcare system, and in this context, reach care and initiate treatment. A systematic review of literature aiming to increase linkage of PLHIV among urban areas of SSA to care and treatment reported four common, active approaches: health system interventions, patient convenience and accessibility, behaviour-related interventions and peer support, and incentives [16]. These active referral methods combat different patient barriers to care and treatment (i.e.; both individual-level barriers–resource-based and cognitive barriers, respectively) [17–19]. Physically assisting patients with reaching their referrals could potentially address multiple psychosocial-level barriers through the provision of an emotional support system, reducing internal stigma and helping the patient cope with their diagnosis [20].

We present a two-phase prospective study from the ZAZI standard of care HIV testing services (HTS) centre providing services for adult clients in Soweto, South Africa. Here, further integrated screenings for NCD markers were performed while also implementing optional assisted linkage to care–accompanied referral to the client’s referral clinic of choice. In this manner, the integrated NCD-HTS model identified both HIV-infection and NCD markers in its clients, and sought to decrease patient attrition in the care cascade across diseases through its active linkage to care. The aim of this study was to compare the proportion of client linkage to care and treatment initiation between the two, independent clinic phases within the same facility: standard of care HTS with passive referral vs integrated NCD-HTS with optional active referral; as well as investigate the time from diagnosis to linkage to care and treatment initiation, and obtain HTS client feedback surrounding the active referral strategy.

## Materials and methods

### Setting

The study was conducted within the ZAZI HTS centre, located at the Perinatal HIV Research Unit (PHRU), a leading research centre within the Wits Health Consortium. The PHRU is affiliated with the University of the Witwatersrand Faculty of Clinical Medicine, situated at Chris Hani Baragwanath Academic Hospital within Soweto, South Africa.

### Study design

This is a two-phase prospective study of a health screening programme at the ZAZI HTS Centre. The programme implemented two different approaches of health screening (standard of care HTS [or baseline] vs integrated NCD-HTS), each with their own mode of patient referral for an abnormal/positive screening result (passive only vs a choice between passive or active referral and linkage to care to the client’s local, referral clinic of choice). Standard of care HTS with passive referral and linkage to care operated from 19 February 2018 through 14 June 2018. Counsellors conducted height, weight, and blood pressure (BP) measurements, sexually transmitted infection (STI) and tuberculosis (TB) symptoms screening, and HIV rapid testing. Integrated NCD-HTS with optional navigated linkage to care immediately followed, from 18 June 2018 through 28 March 2019, within the same clinic space operated by the same healthcare providers. In addition to the standard of care HTS screening, integrated NCD-HTS also provided body mass index (BMI) classifications, rapid testing for both blood glucose (both random and average glycated haemoglobin [HbA1c]) and total cholesterol (TC; via full lipid profile) for all HTS clients; and rapid Chlamydia testing for women. Within integrated NCD-HTS, clients had the option of peer-navigated referral and linkage to care—a clinic staff member accompanying them to their local, referral clinic of choice, with the help of a driver, if needed.

#### Inclusion and exclusion criteria

Eligibility criteria: at least 18 years of age; able to communicate in either English, IsiZulu and/or Sesotho; able and willing to provide written or verbal informed consent (with impartial witness if failed literacy assessment) for both the HTS health screening procedures and for participation in the study-related Linkage to Care Follow-up surveys, and have been referred to the client’s local clinic of choice for at least one abnormal or positive screening result (see: Study Measures: Abnormal Results / Referrals). Only ART-naïve participants were referred for HIV-infection. Each participant had to either own or have access to a mobile or landline telephone and be willing to share that telephonic contact number with study staff for use in study follow-ups.

Any client who consented to either phase of the HTS programme and the use of their data was included in the aggregate data capture.

Exclusion criteria: Immediate need for higher level of care, confirmed TB, and female clients only receiving an abnormal Pap smear result. PHRU’s TB clinic referred confirmed TB patients into the HTS centre for expedited HIV testing to minimise risk of TB exposure to staff and other clients. Therefore, these TB patients were not approached to take part in the study. The HTS centre also performed cervical cancer screening for female clients. If an abnormal Pap smear result was the only abnormal/positive result a female client received, she was excluded from the study, as this screening was not the focus of this study.

#### Sampling and sample size

The study sample was a convenience sample comprised of all eligible (see: Inclusion and exclusion criteria above) walk-in standard of care HTS and integrated NCD-HTS clients presenting to the clinic during the aforementioned timeframe of operation. Clinic hours were Monday through Thursday from 8hrs until 16hrs.

### Data collection and management

Client literacy was assessed through asking each client to read the first paragraph of the HTS programme informed consent. Client demographic questionnaires were either self-collected by literate clients in the reception area or collected with the assistance of clinic staff for illiterate clients. Clinical data were collected by the three study counsellors and one nurse. All referral letters were given to clients by the study nurse, and all client linkage to care follow-up data was collected by counsellors and Linkage Navigators (paid clinic staff managing active linkage to care). All data were collected through questionnaires/surveys and screenings and recorded directly onto paper forms comprising the PHRU HTS client file. Data from client files were cleaned and verified prior to entry into the Research Electronic Data Capture (REDCap) database.

#### Study measures

*Demographics*. Data were collected on sex, age, race, nationality, ethnic group, marital status, highest level of education, source of income, and details regarding home residence–type of housing, source of water and fuel for cooking and lighting, type of toilet facility, as well as possession of household and ownership items.

*Abnormal/positive screening results/referrals*. Standard of care HTS clients were given a referral for care and treatment if they had any of the following: high (>140/90 mmHg) or low (<90/60 mmHg) BP, one or more STI and/or TB symptom(s) according to national syndromic management guidelines [21] (described previously) [22], and/or HIV-infection. These clients then left the HTS clinic to passively link themselves to care.

The referrals for integrated NCD-HTS included standard of care HTS referrals, as well as the following: hyperglycaemia (HbA1c ≥ 6.5 mmol/L and/or random blood glucose ≥ 11 mmol/L), high TC (≥5 mmol/L), and positive rapid Chlamydia test (STI referral). These clients were then given a choice of passive linkage to care or to pair up with a peer-navigator for active linkage to care, organised for either same-day or at a day/time of client’s choice.

*Client linkage to care follow-up survey*. We developed a structured HTS client linkage to care follow-up survey consisting of both closed-ended questions, assessing if the client had made it to their referral(s) and if so, were initiated on treatment; and open-ended questions for free response. The responses to the open-ended questions helped us to further understand the responses to the closed-ended questions (e.g.; why the client refused active linkage to care (if integrated NCD-HTS), the date of linkage to care and treatment initiation, and/or reasons behind not reaching referrals and/or being initiated on treatment).

For standard of care HTS, the first follow-up call was conducted one month after the client obtained his/her referral(s). If there were any outstanding referrals identified as remaining, the follow-up call was repeated for those specific referrals at month two, and then at month three, if needed.

In the case of integrated NCD-HTS, if after the clinic visit, a client chose active linkage to care, the Linkage Navigator made the first follow-up call at the end of that day (i.e.; same day as clinic visit). If at the time of follow-up, there were still outstanding referrals identified as remaining, the HTS client was asked if they would like the help of a Linkage Navigator for the remaining referrals. If so, an appointment for navigated referral was scheduled. With any referrals not having been attended by the client, the survey was repeated at two weeks, one month, two months, and three months, if needed.

*Client linkage to care and treatment initiation*. For the purposes of the study, *successful linkage to care* followed the Centers for Disease Control and Prevention (CDC) definition of, *a newly-identified HIV-infected person engaging with a health care provider within 30 days of diagnosis* (at the ZAZI HTS centre) [16]. This definition was adopted for linkage to care for all conditions–communicable and NCDs, alike–and for *successful treatment initiation*, which was additionally defined as an initiation of pharmacological treatment within 30 days of diagnosis. If a client reported linkage to care and/or treatment initiation between one and three months after diagnosis, they were considered to have *delayed linkage to care* and/or *delayed treatment initiation*. Clients who self-reported they did link to care, and the healthcare practitioner did not initiate them on treatment for a condition, stating they did not need treatment, were categorised as *treatment not needed*. A client for which follow-up contact was not successful (i.e. the HTS client did not answer their mobile phone) or who had not yet reached his or her referral appointment within three months was defined as lost to follow-up.

### Quantitative data analysis

Descriptive statistics (e.g. medians and interquartile ranges as well as means and standard deviation) were determined for continuous variables. Frequencies and their percentages were determined for categorical variables and stratified by phase (Phase 1 and Phase 2) where appropriate.

To test statistical significance for categorical measures stratified by phase, Fisher’s exact or chi-square tests were used. Comparisons for descriptive statistics, such as medians (interquartile ranges) and means (standard deviations) for continuous variables by HTS phase were determined using the Kruskal-Wallis test and Student’s T-Test. The standard of care and integrated NCD HTS (overall, passive, and active) care cascades from client health screenings through initiation of treatment were presented graphically.

All statistical analyses were conducted in SAS Enterprise Guide 7.1 (SAS Institute, Cary, NC) using SAS/STAT procedures.

### Data analysis of open-ended questions

Using SAS Enterprise Guide 7.1 (SAS Institute, Cary NC), a print out of all open-ended responses per survey question was obtained. The first author read through each text response to gain an overall understanding of the data and identify thematic codes. All codes were collated in an excel spreadsheet under the pre-determined themes. If a code did not fit under a pre-determined theme, new themes were created. Categories were then analysed further to create more detailed sub-categories.

The responses in each category were determined by running frequencies. A response to a particular question could be assigned to multiple categories, which may increase the sample size of certain variables. All non-responses to a particular question (free responses left blank by participants) and responses agreed upon as non-logical were not included in the analysis, thus decreasing the sample size for that question. Agreement on the data analysis was achieved through a second review by a co-author.

### Ethical considerations

The ZAZI health programme and embedded study were both approved by the University of Witwatersrand, Human Research Ethics Committee (Wits HREC). The study was reviewed in accordance with the CDC human research protection procedures and was determined to be programmatic research, but CDC investigators did not interact with human subjects or have access to identifiable data or specimens for research purposes (CGH 2018–017). All voluntary participants provided written informed consent prior to any study procedures being conducted.

## Results

**Fig 1:**
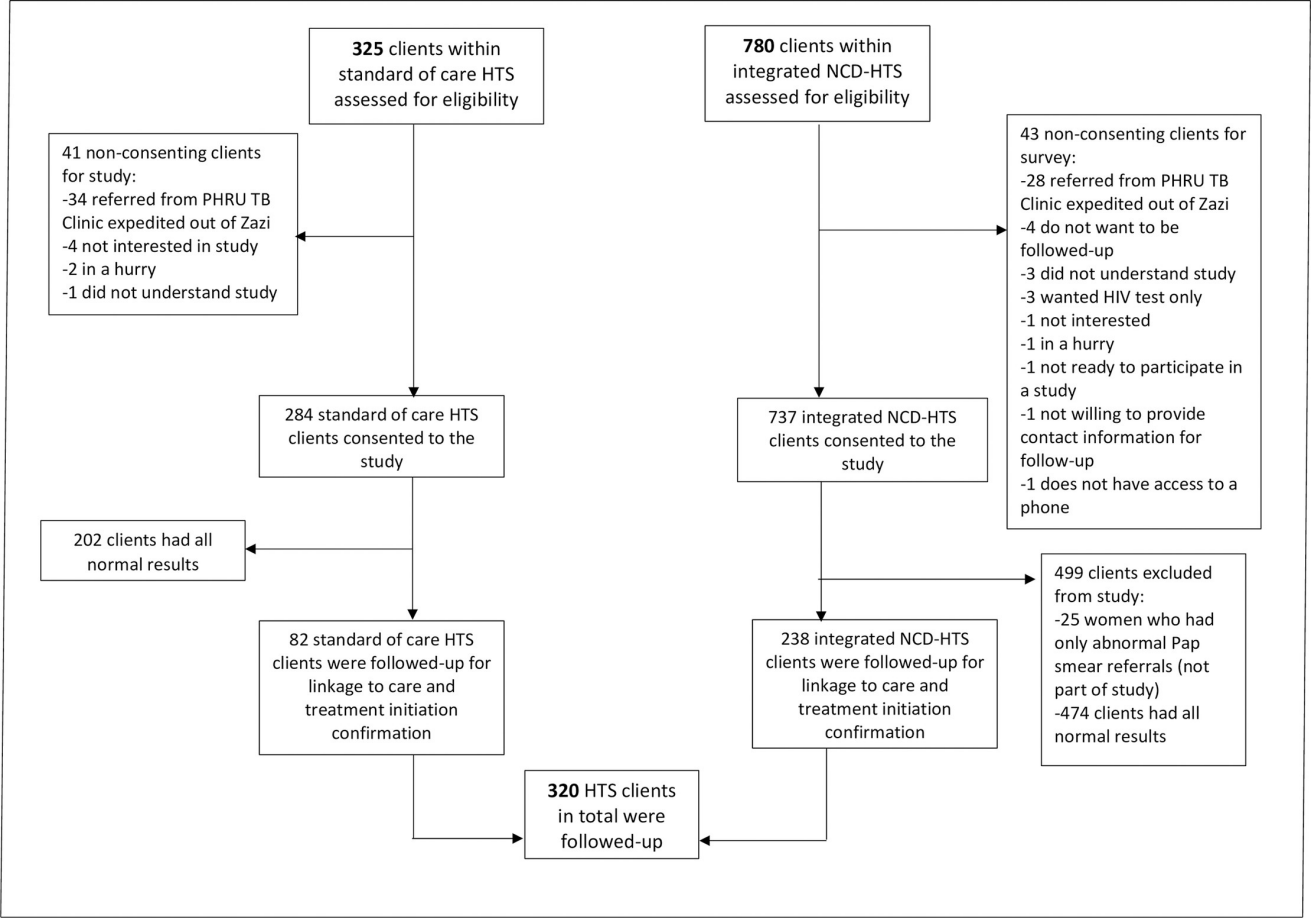
Standard of care HTS vs integrated NCD-HTS participant flow chart, Soweto, South Africa.

Although 1,021 HTS clients consented for follow-up, only 314 individuals were eligible for this study as they had abnormal or positive results that required referral. Of these clients, six participated in both HTS phases, and therefore there were 82 sets of client referrals from the standard of care HTS and 238 client referrals from NCD-HTS for follow-up (N = 320 client referrals). Fig 1 depicts the participant flow from eligible walk-in clients through to enrolled study participants for each phase.

### Demographic and clinical characteristics at initial screening

Of the 314 individual participants, the median age was 37.0 years (interquartile range [IQR]:29.0–44.0), most were female (67.8%, n = 213/314), single (69.7%, n = 219/314), with incomplete high school education (42.2%, n = 132/313), and were neither employed nor self-employed (i.e. income came from social/disability grants, parents, and pensions) (43.2%, n = 135/312). HIV prevalence was high (44.4%, n = 139/313), one third of participants had high BP readings (33.4%, n = 105/313), and 2.2% (n = 7/313) had low BP readings. Standard of care HTS had significantly more participants who were non-South African citizens (8.8%[n = 7/80] vs 1.7%[n = 4/232]; p = 0.0033), divorced/widowed (12.2%[n = 10/82] vs 3.4%[n = 8/232]; p = 0.0034), and who had high BP (50.0%[n = 41/82] vs 27.6%[n = 64/232]; p<0.0001) as compared to integrated NCD-HTS. However, integrated NCD-HTS had significantly more participants who were living together/married than standard of care HTS (27.6%[n = 64/232] vs 15.9%[n = 13/82]; p = 0.0338). Integrated NCD-HTS had more females (70.3%, n = 163/232) and those with incomplete high school education (43.5%, n = 101/232).(Table 1).

**Table 1:** Baseline characteristics of participants by HTS phase at ZAZI HTS centre, Soweto, South Africa.

Variable	Total	Standard HTS &Passive LTC	NCD-HTS & Optional Active LTC	P-Value
**Age (in years)**				
18–24 (%)	43/314 (13.7)	10/82 (12.2)	33/232 (14.2)	0.9642
25–34 (%)	92/314 (29.3)	24/82 (29.3)	68/232 (29.3)	
35–44 (%)	102/314 (32.5)	28/82 (34.1)	74/232 (31.9)	
≥45 (%)	77/314 (24.5)	20/82 (24.4)	57/232 (24.6)	
**Median age (IQR)**	37.0 (29.0–44.0)	37.0 (31.0–43.0)	37.0 (28.0–44.0)	0.5818
**Sex**				
Female (%)	213/314 (67.8)	50/82 (61.0)	163/232 (70.3)	0.1219
Male (%)	101/314 (32.2)	32/82 (39.0)	69/232 (29.7)	
**Race**				
Black African (%)	311/314 (99.0)	80/82 (97.6)	231/232 (99.6)	0.1081
Coloured/White (%)	3/314 (1.0)	2/82 (2.4)	1/232 (0.4)	
**Nationality**				
Other (%)	11/312 (3.5)	7/80 (8.8)	4/232 (1.7)	0.0033
South African (%)	301/312 (96.5)	73/80 (91.3)	228/232 (98.3)	
**Ethnic group**				
Other (%)	43/311 (13.8)	13/81 (16.0)	30/230 (13.0)	0.4587
Sotho (%)	55/311 (17.7)	15/81 (18.5)	40/230 (17.4)	
Tsonga (%)	28/311 (9.0)	6/81 (7.4)	22/230 (9.6)	
Tswana (%)	27/311 (8.7)	11/81 (13.6)	16/230 (7.0)	
Xhosa (%)	32/311 (10.3)	7/81 (8.6)	25/230 (10.9)	
Zulu (%)	126/311 (40.5)	29/81 (35.8)	97/230 (42.2)	
**Marital status**				
Divorced\widowed (%)	18/314 (5.7)	10/82 (12.2)	8/232 (3.4)	0.0034
Living Together\married (%)	77/314 (24.5)	13/82 (15.9)	64/232 (27.6)	0.0338
Single (%)	219/314 (69.7)	59/82 (72.0)	160/232 (69.0)	0.6129
**Highest education**				
No formal education (%)	5/313 (1.6)	1/81 (1.2)	4/232 (1.7)	0.2669
Primary school (%)	14/313 (4.5)	2/81 (2.5)	12/232 (5.2)	
High school (%)	132/313 (42.2)	31/81 (38.3)	101/232 (43.5)	
Matriculated (%)	102/313 (32.6)	25/81 (30.9)	77/232 (33.2)	
Tertiary education (%)	60/313 (19.2)	22/81 (27.2)	38/232 (16.4)	
**Source of money to live on?**				
Employed (%)	115/312 (36.9)	31/82 (37.8)	84/230 (36.5)	0.8070
Self-employed (%)	62/312 (19.9)	15/82 (18.3)	47/230 (20.4)	
Social/Disability grant (%)	45/312 (14.4)	11/82 (13.4)	34/230 (14.8)	
Parents (%)	40/312 (12.8)	10/82 (12.2)	30/230 (13.0)	
Pension (%)	14/312 (4.5)	6/82 (7.3)	8/230 (3.5)	
Unemployed (%)	36/312 (11.5)	9/82 (11.0)	27/230 (11.7)	
**HIV Result**				
Positive (%)	139/313 (44.4)	43/82 (52.4)	96/231 (41.6)	0.0885
Negative (%)	174/313 (55.6)	39/82 (47.6)	135/231 (58.4)	
**Syndromic STI**				
1+ symptoms (%)	38/314 (12.1)	11/82 (13.4)	27/232 (11.6)	0.6716
No symptoms (%)	280/314 (89.2)	71/82 (86.6)	209/232 (90.1)	
**Syndromic TB**				
1+ symptoms (%)	21/314 (6.7)	4/82 (4.9)	17/232 (7.3)	0.4453
No symptoms (%)	293/314 (93.3)	78/82 (95.1)	215/232 (92.7)	
**Blood Pressure**				
High (%)	105/313 (33.4)	41/82 (50.0)	64/232 (27.6)	<0.0001
Low (%)	7/313 (2.2)	4/82 (4.9)	3/232 (1.3)	
Normal (%)	202/313 (64.3)	37/82 (45.1)	165/232 (71.1)	
**Blood glucose**				
High (%)	66/230 (28.7)	-	66/230 (28.7)	-
Normal (%)	164/230 (71.3)	-	164/230 (71.3)	
**Total Cholesterol (TC)**				
High (%)	57/232 (24.6)	-	57/232 (24.6)	-
Low (%)	22/232 (9.5)	-	22/232 (9.5)	
Normal (%)	153/232 (65.9)	-	153/232 (65.9)	

Six Integrated NCD-HTS participants were excluded, as they were also within standard of care HTS.

NB: Of the HIV-infected participants, 9 were not followed up because they were already on treatment, and 2 were of those excluded from the table. Abnormal blood pressure within this table was reported as an average of the two blood pressure readings during screening and not entirely a reflection of true referrals. Of those with high blood glucose, 4 were of those excluded from the table. There was 1 participant with high blood cholesterol who was not followed up.

Human Immunodeficiency Virus (HIV), HIV Testing Services (HTS), Linkage to Care (LTC), Non-communicable disease (NCD), Interquartile range (IQR), Sexually Transmitted Infections (STIs), Tuberculosis (TB).

### Care cascades with time to care and treatment initiation by HTS phase and referral

#### Overall, standard of care referrals from both standard of care and integrated NCD-HTS

Of 320 client referrals across both standard of care and integrated NCD-HTS, 40.0% were referred for HIV-infection, 11.9% for STIs, 6.6% for TB symptoms, and 28.8% for either high or low BP. The majority of these referred clients were linked to care within three months for ART (68.8%, n = 88/128), STIs (73.7%, n = 28/38), TB (71.4%, n = 15/21), and high/low BP (84.8%, n = 78/92) (S1 Table).

It took a median time of 6.5 days (IQR:2.0–19.0), 8.0 days (IQR:3.0–23.0) and 10.0 days (IQR:2.0–20.0) for clients referred for STIs, abnormal BP, and TB, respectively, to link to care for these conditions, overall (Table 2). Of all eligible participants who did link to care across both HTS phases, 88.6% (n = 78/88) were initiated on ART, 78.6% (n = 22/28) were initiated on treatment for STIs, 6.7% (n = 1/15) began TB treatment, and 37.2% (n = 29/78) were initiated on anti-hypertensive medications. Of all referred clients, 60.9% (n = 78/128) were initiated on ART, and 57.9% (n = 22/38) were treated for STIs, 4.8% (n = 1/21) for TB, and 31.5% (n = 29/92) for high BP (S1 Table).

**Table 2:** Successful vs delayed linkage to care and time from referral to care by HTS phase in Soweto, South Africa.

Variable	HIV/ART	TB	STI	BP	Blood glucose	Blood cholesterol
**Total HTS Linkage**						
Successful (%)	75/127 (59.1)	14/21 (66.7)	24/38 (63.2)	65/92 (70.7)	48/70 (68.6)	39/56 (69.6)
Delayed (%)	12/127 (9.5)	1/21 (4.8)	4/38 (10.5)	13/92 (14.1)	9/70 (12.9)	7/56 (12.5)
Lost to follow-up (%)	40/127 (31.5)	6/21 (28.6)	10/38 (26.3)	14/92 (15.2)	13/70 (18.6)	10/56 (17.9)
Median (IQR)	7.0 (2.0–18.0)	10.0 (2.0–20.0)	6.5 (2.0–19.0)	8.0 (3.0–23.0)	7.0 (1.0–21.0)	11.5 (3.0–19.0)
Min, Max	(0–59)	(0–34)	(0–76)	(0–73)	(0–73)	(0–73)
**Standard of Care HTS**						
Successful (%)	19/42 (45.2)	2/4 (50.0)	3/11 (27.3)	21/37 (56.8)	-	-
Delayed (%)	3/42 (7.1)	1/4 (25.0)	2/11 (18.2)	8/37 (21.6)	-	-
Lost to follow-up (%)	20/42 (47.6)	1/4 (25.0)	6/11 (54.6)	8/37 (21.6)	-	-
Median number of days (IQR)	8.0 (2.0–22.0)	10.0 (2.0–34.0)	10.0 (4.0–32.0)	13.0 (6.0–32.0)	-	-
Min, Max	(0–58)	(2–34)	(2–35)	(1–65)	-	-
**Overall Integrated NDC-HTS**						
Successful (%)	56/85 (65.9)	12/17 (70.6)	21/27 (77.8)	44/55 (80.0)	48/70 (68.6)	39/56 (69.6)
Delayed (%)	9/85 (10.6)	-	2/27 (7.4)	5/55 (9.1)	9/70 (12.9)	7/56 (12.5)
Lost to follow-up (%)	20/85 (23.5)	5/17 (29.4)	4/27 (14.8)	6/55 (10.9)	13/70 (18.6)	10/56 (17.9)
Median (IQR)	7.0 (2.0–17.0)	8.0 (1.5–18.5)	6.0 (1.0–17.0)	8.0 (2.0–14.0)	7.0 (1.0–21.0)	11.5 (3.0–19.0)
Min, Max	(0–59)	(0–27)	(0–76)	(0–73)	(0–73)	(0–73)
**Integrated NCD-HTS Passive Linkage**						
Successful (%)	47/72 (65.3)	7/12 (58.3)	20/24 (83.3)	41/51 (80.4)	41/60 (68.3)	34/51 (66.7)
Delayed (%)	8/72 (11.1)	-	2/24 (8.3)	5/51 (9.8)	8/60 (13.3)	7/51 (13.7)
Lost to follow-up (%)	17/72 (23.6)	5/12 (41.7)	2/24 (8.3)	5/51 (9.8)	11/60 (18.3)	10/51 (19.6)
Median number of days (IQR)	8.0 (3.0–19.0)	17.0 (2.0–20.0)	6.5 (2.0–17.0)	8.0 (2.0–14.0)	7.0 (1.0–21.0)	11.0 (3.0–19.0)
Min, Max	(1–59)	(1–24)	(0–76)	(0–73)	(0–73)	(1–73)
**Integrated NCD HTS Active Linkage**						
Successful (%)	9/13 (69.2)	5/5 (100.0)	1/3 (33.3)	3/4 (75.0)	7/10 (70.0)	5 /5(100.0)
Delayed (%)	1/13 (7.7)	-	-	-	1/10 (10.0)	-
Lost to follow-up (%)	3/13 (23.1)	-	2/3 (66.7)	1/4 (25.0)	2/10 (20.0)	-
Median number of days (IQR)	0.0 (0.0–6.0)	6.0 (0.0–10.0)	0.0 (0.0)	6.0 (0.0–15.0)	3.5 (0.0–23.5)	12.0 (10.0–15.0)
Min, Max	(0–49)	(0–27)	(0)	(0–15)	(0–49)	(0–27)

NB: One referred participant was excluded from HIV/ART due to missing date of linkage to care.

*Successful linkage to care* was defined as have been linked to care within 30 days (one month); *Delayed linkage to care* was defined as having been linked to care between one and three months; *Lost to follow-up* is defined as not having confirmed linkage to care by three months. There were no significant differences in time from referral to linkage to care for any referral type between HTS phases, overall. There were significantly more NCD-HTS participants who chose passive linkage who achieved successful linkage to care for STI referrals as compared to those who chose active linkage (83.3%[n = 20/24] vs 33.3%[n = 1/3]; p = 0.0495.

Human Immunodeficiency Virus (HIV), Antiretroviral Therapy (ART), Tuberculosis (TB), Sexually Transmitted Infections (STIs), Blood Pressure (BP), Interquartile Range (IQR), Non-communicable Disease (NCD), HIV Testing Services (HTS).

Across HTS phases, it took an average of 2.0 days (IQR:2.0–2.0; for TB) and 9.0 days (IQR:5.0–21.0; for ART) for clients to initiate treatment (Table 3). Treatment was not needed for a high proportion of those linked to care for high/low BP in either HTS Phase (standard of care HTS: 58.6%[n = 17/29]; integrated NCD-HTS:61.2%[n = 30/49]).(Table 3) None of the participants referred for low BP were initiated on treatment across HTS phases.

**Table 3:** Successful vs delayed treatment initiation and time from referral to treatment initiation by HTS phase in Soweto, South Africa.

Variable	HIV/ART	TB	STI	BP	Blood glucose	Blood cholesterol
**Total HTS Linkage**						
Successful (%)	68/88 (77.3)	1/3 (33.3)	18/28 (64.3)	13/78 (16.7)	13/57 (22.8)	14/46 (30.4)
Delayed (%)	10/88 (11.4)	-	4/28 (14.3)	16/78 (20.5)	1/57 (1.8)	2/46 (4.4)
Treatment not needed (%)		-	6//28 (21.4)	47/78 (60.3)	40/57 (70.2)	29/46 (63.0)
Lost to follow-up (%)	10/88 (11.4)	2/3 (66.7)	-	2/78 (2.6)	3/57 (5.3)	1/46 (2.2)
Median (IQR)	9.0 (5.0–21.0)	2.0 (2.0–2.0)	6.5 (2.0–28.0)	8.0 (2.0–18.0)	14.5 (7.0–24.0)	11.5 (1.5–21)
Min, Max	(0–70)	(2–2)	(0–76)	(0–63)	(1–35)	(0–46)
**Standard of Care HTS**						
Successful (%)	17/22 (77.3)	1/3 (33.3)	3/5 (60.0)	9/29 (31.0)	-	-
Delayed (%)	3/22 (13.6)	-	2/5 (40.0)	2/29 (6.9)	-	-
Treatment not needed (%)	-	1/3 (33.3)	-	17/29 (58.6)	-	-
Lost to follow-up (%)	2/22 (9.1)	1/3 (33.3)	-	1/29 (3.5)	-	-
Median number of days (IQR)	19.5 (6.5–26.5)	2.0 (2.0–2.0)	10.0 (4.0–32.0)	8.0 (2.0–29.0)	-	-
Min, Max	(1–57)	(2–2)	(2–35)	(1–63)	-	-
**Overall Integrated NCD-HTS Linkage**						
Successful (%)	51/66 (77.3)	-	15/23 (65.2)	17/49 (34.7)	13/57 (22.8)	14/46 (30.4)
Delayed (%)	7/66 (10.6)	-	2/23 (8.7)	1/49 (2.0)	1/57 (1.8)	2/46 (4.4)
Treatment not needed (%)	-	-	6/23 (26.1)	30/49 (61.2)	40/57 (70.2)	29/46 (63.0)
Lost to follow-up (%)	8/66 (12.1)	-	-	1/49 (2.0)	3/57 (5.3)	1/46 (2.2)
Median (IQR)	8.0 (5.0–17.0)	-	5.0 (1.0–21.0)	8.0 (2.0–13.0)	14.5 (7.0–24.0)	11.5 (1.5–21.0)
Min, Max	(0–70)	-	(0–76)	(0–33)	(1–35)	(0–46)
**Integrated NCD-HTS Passive Linkage**						
Successful (%)	42/56 (75.0)	-	14/22 (63.6)	15/46 (32.6)	10/49 (20.4)	11/41 (26.8)
Delayed (%)	6/56 (10.7)	-	2/22 (9.1)	1/46 (2.2)	1/49 (2.0)	2/41 (4.9)
Treatment not needed (%)	-	-	6/22 (27.3)	29/46 (63.0)	35/49 (71.4)	28/41 (68.3)
Lost to follow-up (%)	8/56 (14.3)	-	-	1/46 (2.2)	3/49 (6.1)	-
Median number of days (IQR)	9.0 (6.0–17.5)	-	6.5 (1.5–24.5)	8.5 (3.0–16.0)	13.0 (5.0–21.0)	11.0 (2.0–19.0)
Min, Max	(1–70)	-	(1–76)	(0–33)	(1–35)	(1–46)
**Integrated NCD-HTS Active Linkage**						
Successful (%)	9/10 (90.0)	-	1/1 (100.0)	2/3 (66.7)	3/8 (37.5)	3/5 (60.0)
Delayed (%)	1/10 (10.0)	-	-	-	-	-
Treatment not needed (%)	-	-		1/3 (33.3)	5/8 (62.5)	1/5 (20.0)
Lost to follow-up (%)	-	-	-	-	-	1/5 (20.0)
Median number of days (IQR)	2.5 (0.0–8.0)	-	0.0 (0.0)	3.0 (0.0–6.0)	24.0 (11.0–27.0)	12.0 (0.0–27.0)
Min, Max	(0–49)	-	(0)	(0–6)	(11–27)	(0–27)

NB: One referred participant was excluded from HIV/ART due to missing date of linkage to care.

*Successful treatment initiation* was defined as having initiated treatment within 30 days (one month); *Delayed initiation of treatment* was defined as having initiated treatment between one and three months; *Lost to follow-up* was defined as not having confirmed treatment initiation by three months. Treatment not needed did not exclude prescribed lifestyle modification.

The time in days from referral to treatment initiation was shorter in integrated NCD-HTS relative to standard of care HTS for ART [12.8 (SD = 13.9) vs. 17.8 (SD = 13.9); p = 0.1762] and BP [9.5 (SD = 9.7) vs. 18.1 (SD = 22.6); p = 0.2543]. It took significantly less number of days for participants who were linked to care for ART to be initiated on treatment in integrated NCD-HTS as compared to standard of care HTS [0.98 (SD = 2.5) vs. 5.84 (SD = 9.0); p = 0.0319]. Human Immunodeficiency Virus (HIV), Antiretroviral Therapy (ART), Sexually Transmitted Infections (STI), Tuberculosis (TB), Blood Pressure (BP), Interquartile Range (IQR), Non-communicable disease (NCD), HIV Testing Services (HTS).

#### Standard of care HTS vs overall integrated NCD-HTS

**Fig 2:**
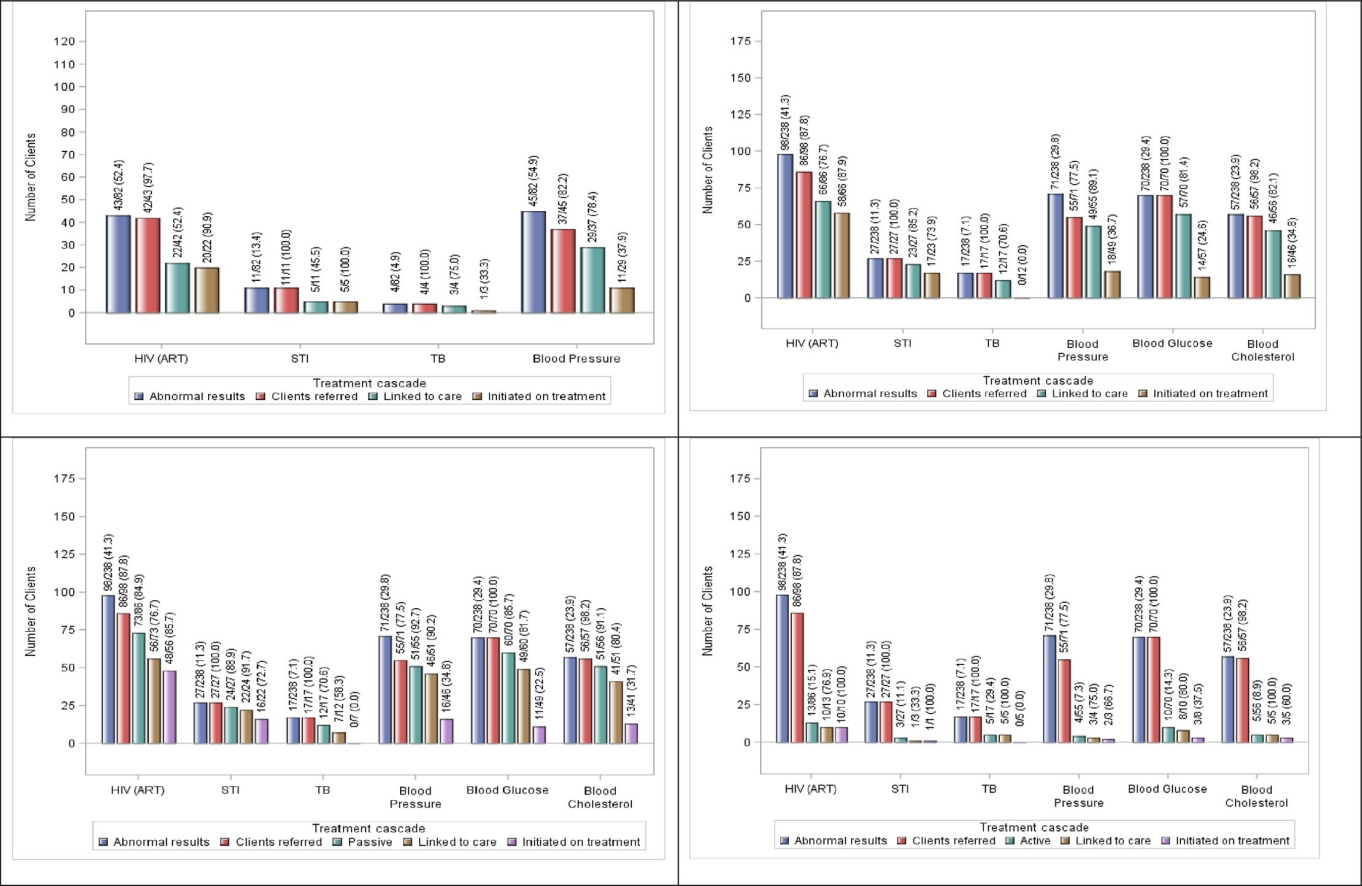
Standard of care HTS and integrated NCD-HTS care and treatment cascades in Soweto, South Africa. (A: Top Left) Standard of Care HTS Cascade with Passive Linkage to Care; (B: Top Right) Overall Integrated NCD-HTS Cascade; (C: Lower Left) Integrated NCD-HTS Cascade with Passive Linkage to Care; (D: Lower Right) Integrated NCD-HTS Cascade with Active Linkage to Care. Only HIV-infected clients who were not already initiated on ART were referred. Abnormal results for BP took the average of two readings, while clients were referred for two abnormal readings. One integrated NCD-HTS client with high cholesterol was not referred. Significantly more clients referred for ART and STIs were linked to care in NCD-HTS as compared to standard of care HTS (76.7%[n = 66/86] vs. 52.4%[n = 22/42], p = 0.0052; and 85.2%[n = 23/27] vs. 45.5%[n = 5/11], p = 0.0117; respectively). Significantly more Phase 2 clients chose passive referral over active referral (89.1% [n = 212/238] vs. 10.9% n = 26/238]; p<0.0001). Of the STI referrals, significantly more clients were linked to care passively as compared to actively (91.7% [n = 22/24] vs. 33.3% [n = 1/3]; p = 0.0073). Sexually Transmitted Infections (STIs), Tuberculosis (TB), Human Immunodeficiency Virus (HIV), Antiretroviral Therapy (ART).

Overall, integrated NCD-HTS–with its optional active referral—had a larger proportion of clients linked to care for HIV, STIs, and high/low BP; with significantly more referred clients linked to care for ART and STIs as compared to standard of care HTS (76.7%[n = 66/86] vs. 52.4%[n = 22/42], p = 0.0052; and 85.2%[n = 23/27] vs. 45.5%[n = 5/11], p = 0.0117; respectively; Fig 2A & 2B). Overall, integrated NCD-HTS also linked clients to care within a shorter time on average, across all conditions, as compared to standard of care HTS (the shortest being 6.0 days for STIs [IQR:1.0–17.0] with the longest being both 8.0 days for TB [IQR:1.5–18.5] and 8.0 days for high/low BP [IQR:2.0–14.0] vs the shortest being 8.0 days for HIV [IQR:2.0–22.0] and the longest being 13.0 days for high/low BP [IQR:6.0–32.0]).(Table 2)

Standard of care HTS–with passive referral, only—had higher proportions of clients who were linked to care subsequently initiated on treatment for each condition as compared to integrated NCD-HTS (Fig 2A and 2B). However, integrated NCD-HTS initiated its clients on treatment in a shorter amount of time for HIV, STIs, and BP compared to standard of care HTS—the shortest time being 5.0 days for STIs [IQR:1.0–21.0] and the longest time being both 8.0 days for ART [IQR:5.0–17.0] and 8.0 days for high BP [IQR:2.0–13.0] vs the shortest time being 8.0 days for high BP [IQR:2.0–29.0] and the longest time being 19.5 days for ART [IQR:6.5–26.5].

For the referrals specific to integrated NCD-HTS, 81.4% (n = 57/70) and 82.1% (n = 46/56) of clients referred for hyperglycaemia and high TC were linked to care; and of those, 24.6% (n = 14/57) and 34.8% (n = 16/46) were initiated on treatment, respectively (Fig 2B). It took 14.5 days on average (IQR:7.0–24.0) for clients to initiate treatment for hyperglycaemia and 11.5 days on average (IQR:1.5–21.0) for high TC. A high proportion of clients did not need treatment once re-assessed at the referral clinic for each condition– 70.2% (n = 40/57) of referred clients for high glucose and 63.0% (n = 29/46) of referred clients for high cholesterol, respectively (Tables 2 and 3).

#### Integrated NCD-HTS passive vs active linkage to care and treatment

Fig 2C and 2D show the integrated NCD-HTS care and treatment cascade for referred clients electing either passive or active linkage to care and treatment.

Significantly more participants chose passive referral over active referral (89.1% [n = 212/238] vs. 10.9% [n = 26/238]; p<0.0001). Of the participants referred for STIs who were linked to care, significantly more chose passive referral as compared to active referral (91.7% [n = 22/24] vs. 33.3% [n = 1/3]; p = 0.0073). There were no other statistically significant differences between the proportions of clients passively and actively linked to care and subsequently initiated on treatment (S2 Table).

Across conditions, a high proportion of integrated NCD-HTS participants were passively linked to care, ranging from 91.7% (n = 22/24) for STIs to 58.3% (n = 7/12) for TB. Those linked to care for STI were linked within the lowest median number of days of 6.5 (IQR: 2.0–17.0), whereas those reaching care for TB did so within the highest median number of days (17.0, IQR: 2.0–20.0) (Table 2).

The majority of integrated NCD-HTS clients who were passively linked to care for ART and did reach care were initiated on treatment successfully (75.0%, n = 42/56) within a median number of days of 9.0 (IQR:6.0–17.5). STI passive referrals also had a high number of participants successfully initiated on treatment (63.6%, n = 14/22). A large proportion of passively referred clients within integrated NCD-HTS did not need to initiate treatment after further assessment: 63.0% (n = 29/46) for BP clients, 71.4% (n = 35/46) and 68.3% (n = 28/41) of clients with hyperglycaemia and high blood cholesterol, respectively. Of the 10 participants who were actively linked to care for ART within integrated NCD-HTS, 90.0% were successfully initiated on treatment in a median of 2.5 (IQR:0.0–8.0) days. (Table 3)

### Reasons for choosing passive over active linkage to care

There were 67 responders who shared 70 total reasons as to why they chose passive referral over active referral (n = 67/212, 31.6%). The main reason stated was that the client preferred to go alone (55.7%). Other reasons described were the close proximity and/or familiarity of the referral clinic (11.4%) and choosing to self-support (8.6%) (e.g.; ‘*I believe I have to take responsibility and go alone’* and *‘I will [have to] go to the clinic [alone] anyway for my treatment’*). Having one’s own transport (7.1%) and/or support system (5.7%) (e.g.; the support of a partner, family member, friend, or work colleague), and wanting privacy or having a referral letter to present to the clinic (2.9%, each) were other reasons for choosing passive linkage to care. Overall, 5.7% expressed they did not like the idea of navigated linkage to care.

### Reasons for not successfully linking to care

There were 75 responses received by clients across both HTS phases yielding 95 reasons for not successfully linking to care across all referrals. Of the 95 reasons, 41.1% were due to being too busy to attend the referral but were still planning on going and 23.2% were not yet ready or needed to absorb their results prior to seeking care and/or treatment services. Other clients were unable to attend their referrals due to various reasons (i.e.; having been sick or hurt, having relocated or been travelling, not having enough money for transport to the clinic, or due to personal and/or family issues; 14.7%). There were blunt refusals towards obtaining treatment from clients referred for HIV-infection and/or high BP, (8.4%). One client referred for high BP shared: *‘[I] never went to the clinic and [I] will not go*. *[I am] not ready to start treatment at the clinic*, *they'd want to [initiate me]*’. Some clients wished to wait for their partner to participate in couples’ HIV counselling and testing before seeking treatment (4.2%).

There were a few barriers (3.2% in total) encountered at the point of entry into the health system (i.e.; not receiving help due to results not being looked at by the provider, having run out of time at the clinic, and the nurse not understanding the referral letter).For example, one client shared: ‘*[I] gave the nurse the referral letter*, *but they only focused on the BP and Cholesterol [referrals and] didn't check [my] STI symptoms’*. Another client shared: ‘*[I] didn't have enough time to be checked for TB when [I] went to the clinic for [my] STI [referral] as [I] had to go to work’*.

Two responses from clients referred for hyperglycaemia and high blood cholesterol shared that they chose to drink traditional African medicine (i.e.; *muti*), instead. Other responses regarded (STI) symptoms having cleared up, forgetting about their referral, and stigma-related reasons serving as a deterrent for linking to care (1.1%, each). For example, one client referred for ART shared: ‘*[I do not] have money to go to the clinic and [I am] not comfortable… because there is family member [there] and [the] clinic is [in my] hood so [I am] comfortable but [I am] making a plan’*.

### Reasons for not being initiated on treatment

There were 160 responses received by clients across both HTS phases yielding 196 reasons for not initiating treatment across all referrals. Of the 196 reasons, 49.7% were due to BP, blood glucose and/or blood cholesterol having normalised and/or receiving a negative STI and/or TB test result upon being re-checked at the referral clinic. For NCD risk factor referrals (i.e.; BP, blood glucose and cholesterol), 16.2% were prescribed a healthy lifestyle modification rather than medication, [‘*Client went to the clinic*, *but they didn't initiate her*. *They said she must boost her iron by changing her diet and eat[ing] more of the food that* boosts *iron*.’]; 14.7% were to undergo continued monitoring; and 6.1% were already initiated on treatment prior to their diagnosis.

Other reasons for non-initiation were that laboratory tests were conducted (e.g.; blood and/or sputum analysis), administrative tasks had to be completed (e.g.; ART adherence classes), and follow-up appointments were required prior to initiation of treatment (6.1%). For example, three of these clients who had been referred for ART shared similar reasons as follows: [‘*She was given a date to come back after blood was collected*, *but [she] hasn't [gone] back due to hectic work schedule’]*. A fourth HIV-infected client shared: [‘…*[I am] still attending adherence classes and still [need] to go back when free at work*…’].

Additionally, 4.6% were either not helped due to the clinic being full or just not having their condition checked, [‘*I couldn't open file*, *because the clinic was full*.’] and [‘*They said they won't give me treatment*, *because it is caused by stress—even when it was 168 over 110*.’]; 1.0% were given an initial pill to control their high BP and/or hyperglycaemia, not a prescription, [‘*The tests were not high enough for him to be initiated on treatment*, *but they gave him a pill to help stabilize the sugar and will continue monitoring*.’]; and one client (0.5%) self-reported that she could not receive STI treatment as she was found to be pregnant.

## Discussion

Our study presents a comparison between standard of care HTS with passive referral and integrated NCD-HTS with optional navigated (active) referral. We have identified that there is still immense fallout within the treatment cascade from referral to care and treatment across diseases, regardless of the referral mode. Despite policies advocating same day ART initiation, the time from referral to treatment initiation was found to span an average of nine days; and for other conditions, time to initiation ranged from an average of two to 14.5 days. The linkage to care of STIs, TB and BP was high despite lower treatment initiation. Significantly more referred patients preferred the standard of care passive referral method as compared to assisted referral. Regardless, having active referral as an option did aid in linking significantly more HIV-infected clients to care than solely offering passive referral. However, this strategy did not translate to significantly more referred PLHIV initiated on ART. We identified individual-level barriers and failures at the health systems point of entry which barred efficient initiation of treatment across diseases, inclusive of the public healthcare sector improperly implementing its healthcare policies and/or guidelines.

Out of all HIV-infected study participants, two-thirds were linked to care and three-fifths were initiated on ART. Our study corroborates other research from sub-Saharan Africa, where it is reported that as much as 40% of PLHIV who are diagnosed are not linked to treatment services [23–25]. South Africa was among the first countries in Africa to adopt the WHO’s Universal Test and Treat (UTT) policy–offering all persons diagnosed with HIV-infection initiation of treatment irrespective of CD4+ count [26], introducing the policy in-country in September 2016 [27]. Furthermore, same-day initiation (SDI), advocating for ART initiation on the day of HIV diagnosis, followed exactly one year later. Three years later, despite these initiatives, our study shows only three-fifths of PLHIV have entered care and started ART, and other data depicts late treatment initiation has not decreased over the past decade [2, 28, 29].

While some HIV-infected participants did achieve SDI of ART, the overwhelming majority did not. This study identified individual and health systems barriers which may be associated with treatment initiation for both HIV and NCD risk factors, thus contributing to attrition within both the HIV and NCD care cascades.

Half of our study participants who didn’t link to care across diseases either actively or passively refused treatment (e.g.; refusing their referral letter vs sharing they had been too busy to attend clinic for various reasons); while one-quarter were not ready to engage with treatment at diagnosis. While our study did not collect further information as to why clients were not treatment-ready, previous studies have shown that patients’ perceived health and the perception that one does not need to start treatment without the presence of symptoms has been a long contributing reason for ART treatment refusal amongst various HIV-infected populations (i.e.; serodiscordant couples, pregnant women, men who have sex with men, etc.) [30–38]. This may also be true for patients with referred NCD risk factors, as many conditions do not present any symptoms (i.e.; hypertension, hyperglycaemia, hypercholesterolemia) [39–41]. Chronic diseases (both HIV and NCDs, alike) require daily adherence of medication, and perceived treatment regimen demands and side effects are known deterrents within populations of lower income with poor health literacy [42].

Our study participants who did link to care with the intention of initiating treatment still experienced health system barriers which may have negatively affected treatment initiation at the point of care: non-adherence of healthcare providers to current treatment policies/guidelines and an overburdened health care system.

Despite the UTT and SDI policies, our study provides evidence of PLHIV having additional blood testing conducted (including CD4 count) and/or being required to complete three ART adherence counselling modules prior to initiating treatment at a scheduled follow-up appointment. We also identified stage 2 hypertensive patients who were not initiated on medication as per national guidelines [43], and/or patients with uncontrolled NCD risk factors (i.e.; had already initiated treatment, but remained with abnormal screening results) who may not have had their current treatment regimens re-assessed and/or adjusted for optimal health outcomes. A large proportion of our clients with NCD risk factors self-reported they were told they ‘did not need treatment [with pharmaceuticals]’ after being re-assessed at the referral clinic after successfully linking to care. Over two-thirds of referred clients for abnormal BP, cholesterol and glucose were told they did not need to initiate treatment. It is possible that the confirmatory testing methods used at referral clinics differed from those used at the ZAZI HTS Centre, yielding different results (e.g.; fasting plasma glucose testing vs our average blood glucose testing). Additional research would be needed to determine if this was true, and if so, a reassessment of standard of care protocols within the public health sector is critical. Numerous specialist committees on chronic disease management support the earlier screening and treatment of disease, as timely initiation of treatment optimises health outcomes and is cost-effectiveness in the long-term, saving on future costs relating to chronic disease-related morbidity and mortality [9].

Additionally, our clients’ self-reported reasons eluded to the overburdened health care system as still being a deterrent to treatment initiation. Many of our study participants cited they were not helped due to the clinic being too full, or if they were seen by a healthcare provider for comorbidities, some conditions deemed less critical (i.e.; cholesterol) were not checked or addressed as the provider did not have enough time. Not only are each of these cases missed opportunities for intervention, which would decrease the likelihood of morbidity and mortality, but the patient must have continued mental resilience to re-engage with the healthcare system that failed them [44, 45].

While a known challenge towards achieving rapid linkage to care and initiation of treatment is passive referral [15], our study identified yet another challenge–significantly more referred study participants actually prefer passive as compared to assisted linkage to care, with most participants sharing they simply preferred to go alone. Perhaps the reasoning could more truly be related to experienced internal and fear of external stigma, which drives the desire to be alone. Studies have found internalised shame felt by people living with disease can create self-defeating beliefs, leading to demoralization and the desire to conceal one’s status for fear of social isolation [34]. Furthermore, an emerging theme of a scoping literature review depicted negative patient perceptions of healthcare providers–largely the fear of being mistreated due to stigma [46]—within HIV platforms may also have a negative impact on the engagement with NCD care, either located within or outside of HIV platforms, and therefore effect the NCD-care continuum [47]. There were also HTS clients who refused peer-navigated referral and linkage to care due to the preference for self-supporting and taking responsibility for themselves. Some PLHIV may simply feel they are capable of managing their own care and health, and studies have shown a perception of one’s own role and responsibility in maintaining one’s own health is correlated with better ART adherence [48].

Even though passive linkage to care was preferred, active linkage to care did link significantly more HIV-infected patients to care and in a shorter amount of time on average. Our active referral provided a support system, which creates a sense of humanity and belonging, and is known to decrease stigmatised beliefs [34, 49]. A study amongst HIV-infected adolescents and youth in Kenya, showed significant improvements in linkage to and early retention in care after the implementation of peer-navigated services and psychosocial support were offered [50]. A systematic review and meta-analysis of community and facility-based HIV testing and linkage to care gaps in sub- Saharan Africa reported that facilitated or ‘*active*’ linkage services achieved both higher linkage to care and ART initiation than passive referral [51]. The same could be described for NCD linkage to care. A study among referred hypertensive patients in rural Uganda that included an intervention package consisting of a short counselling session after diagnosis, a referral appointment within 30 days, and a transportation voucher, resulted in 83% linkage to care [52].

Our study stresses that active linkage to care does indeed work. It then becomes important for the public healthcare sector to emphasise a case managing approach through community health workers or case managers and focus on any health systems issues affecting rapid treatment initiation. Dedicated staff members can forge close relationships with their patients, working through individual-level health-seeking behavioural barriers and supporting the patient’s journey from diagnosis of disease through initiation of treatment and treatment adherence (even to achievement of viral load suppression for HIV-infected patients).

### Limitations

This study was a cross-sectional study and may not explain causality. This study was a direct comparison, which is descriptive, and therefore, there was no control for any parameters. Results could be attributable to other study-related aspects—potentially bias of populations attending the facility at different times. Study enrolment was not designed to discriminate between these different groups and it was purely a random process. Bias may have also been introduced due to the additional screenings in the integrated HTS where more time was required and there was a larger sample size—possibly having resulted in more HIV-infected clients referred with more than one condition. Patients with multiple chronic conditions tend to use services more frequently [53]. While we conducted this study successfully in our setting, more research would be required within the public health facilities to get data that can be generalized.

The standard of care HTS and integrated NCD-HTS populations had slightly different characteristics, which may have limited the ability to compare them. For example, during integrated NCD-HTS, the HTS clinic was assisting with HIV rapid test screening for the enrolment of women into HIV prevention (pre-exposure prophylaxis) trials conducted at PHRU, as well as conducting screenings for cervical cancer and human papilloma virus (HPV) for female clients. Although there was not a statistically significant difference in proportions of clients by sex between the two HTS phases, this may have encouraged the higher volumes of women attending the clinic during the integrated NCD-HTS phase, as the sex ratio presented within the standard of care phase is a more typical representation.

Some sample sizes for comparisons were small, specifically the sample size for those opting for active linkage to care, which may have affected statistical impact.

The integrated HTS group received more support in the form of two additional telephone calls (same day as screening and two weeks post-screening), which may have increased bias towards better outcomes. Additionally, our reports of linkage to care and treatment initiation were self-reported by the clients and not able to be confirmed through other sources. Furthermore, the study was conducted at a single study site located in an urban settlement with good access to referral sites. The findings may be different in rural or peri-urban settlements.

We were unable to confirm linkage to ART clinics using TIER.net, an accessible electronic database managed by government ART clinic staff to capture data elements and indicators required to monitor HIV and ART services. Additionally, a proportion of clients were lost to follow-up, which may have caused an underestimation or overestimation of reported linkage to care and treatment initiation.

## Conclusions

In resource-limited settings, such as sub-Saharan Africa, it is reported that as much as 40% of PLHIV who are diagnosed are not linked to treatment services [23–25, 29]. This is not unique to HIV programmes, but also for other chronic diseases. Patients who are linked to care, cycle in and out of care with varying levels of adherence [24]. Cost-effective strategies are required to improve linkage to and retention in care and treatment services for people diagnosed with chronic disease. While prompt linkage to treatment and care is ideal and should be encouraged, strategies need to also address various, complex levels of psychosocial and health systems barriers at the point of entry into care and treatment [54]. Perhaps healthcare provider case management approaches should be emphasised. Further research on systems integration within a multi-site study design conducted within public health sector HTS clinics across South Africa is required to provide evidence-based data to support NCD-HTS integrated screening and referral and best strategies for rapid linkage to care and treatment initiation. This would assist in determining the generalisability of these study results and properly assessing the transferability of such health systems strengthening strategies.

## Supporting information

S1 Table Linkage to care and treatment initiation by HTS phase in Soweto, South Africa. HIV Testing Services (HTS), Linkage to Care (LTC), Human Immunodeficiency Virus (HIV), Antiretroviral Therapy (ART), Sexually Transmitted Infections (STI), Tuberculosis (TB), Blood Pressure (BP), Non-communicable disease (NCD).
(DOCX)

S2 Table Comparing passive and active treatment initiation by HTS phase in Soweto, South Africa. Significantly more clients chose passive referral over active referral (89.1% [n = 212/238] vs. 10.9% n = 26/238]; p<0.0001). Non-communicable diseases (NCDs), HIV Testing Services (HTS), Human Immunodeficiency Virus (HIV), Sexually Transmitted Infections (STI), Tuberculosis (TB), Blood Pressure (BP).
(DOCX)

S1 Dataset Dataset used for the study. (ZIP)
