# Supplementary material for: Does peer-navigated linkage to care work? A cross-sectional study of active linkage to care within an integrated non-communicable disease-HIV testing centre for adults in Soweto, South Africa
Source: PLoS One. 2020 Oct 22;15(10):e0241014. doi: 10.1371/journal.pone.0241014 (PMC7580918; doi:10.1371/journal.pone.0241014)
Supplement: S1 Table — HIV Testing Services (HTS), Linkage to Care (LTC), Human Immunodeficiency Virus (HIV), Antiretroviral Therapy (ART), Sexually Transmitted Infections (STI), Tuberculosis (TB), Blood Pressure (BP), Non-communicable disease (NCD). (DOCX) [file pone.0241014.s001.docx]

|  | **Linkage to Care** | | | | **Treatment Initiation** | | | |
| --- | --- | --- | --- | --- | --- | --- | --- | --- |
| **Variable** | **Total** | **Standard HTS & passive LTC** | **NCD-HTS & optional active LTC** | **P-Value** | **Total** | **Standard HTS &**  **passive LTC** | **NCD-HTS & optional active LTC** | **P-Value** |
|  |  |  |  |  |  |  |  |  |
| **HIV/ART (%)** | 88/128 (68.8) | 22/42 (52.4) | 66/86 (76.7) | 0.0052 | 78/88 (88.6) | 20/22 (90.9) | 58/66 (87.9) | 0.6981 |
| **STI (%)** | 28/38 (73.7) | 5/11 (45.5) | 23/27 (85.2) | 0.0117 | 22/28 (78.6) | 5/5 (100.0) | 17/23 (73.9) | 0.1976 |
| **TB (%)** | 15/21 (71.4) | 3/4 (75.0) | 12/17 (70.6) | 0.8605 | 1/15 (6.7) | 1/3 (33.3) | 0/12 (0.0) | - |
| **BP (%)** | 78/92 (84.8) | 29/37 (78.4) | 49/55 (89.1) | 0.1607 | 29/78 (37.2) | 11/29 (37.9) | 18/49 (36.7) | 0.9159 |
|  |  |  |  |  |  |  |  |  |
