# Supplementary material for: Does peer-navigated linkage to care work? A cross-sectional study of active linkage to care within an integrated non-communicable disease-HIV testing centre for adults in Soweto, South Africa
Source: PLoS One. 2020 Oct 22;15(10):e0241014. doi: 10.1371/journal.pone.0241014 (PMC7580918; doi:10.1371/journal.pone.0241014)
Supplement: S2 Table — Significantly more clients chose passive referral over active referral (89.1% [n = 212/238] vs. 10.9% n = 26/238]; p<0.0001). Non-communicable diseases (NCDs), HIV Testing Services (HTS), Human Immunodeficiency Virus (HIV), Sexually Transmitted Infections (STI), Tuberculosis (TB), Blood Pressure (BP). (DOCX) [file pone.0241014.s002.docx]

|  |  | **Confirmed Linkage to Care** | |  | **Confirmed Initiation of Treatment** | |  |
| --- | --- | --- | --- | --- | --- | --- | --- |
| **Variable** | **Referred** | **Passive** | **Active** | **P-Value** | **Passive** | **Active** | **P-Value** |
|  |  |  |  |  |  |  |  |
| **Care & Treatment** |  |  |  |  |  |  |  |
| HIV/ART (%) | 86/238 (36.1) | 56/73 (76.7) | 10/13 (76.9) | 0.9868 | 48/56 (85.7) | 10/10 (100.0) | 0.2057 |
| STI (%) | 27/238 (11.3) | 22/24 (91.7) | 1/3 (33.3) | 0.0073 | 16/22 (72.7) | 1/1 (100.0) | 0.5436 |
| TB (%) | 17/238 (7.1) | 7/12 (58.3) | 5/5 (100.0) | 0.1316 | - | - | - |
| BP (%) | 55/238 (23.1) | 46/51 (90.2) | 3/4 (75.0) | 0.3479 | 16/46 (34.8) | 2/3 (66.7) | 0.2670 |
| Blood Glucose (%) | 70/238 (29.4) | 49/60 (81.7) | 8/10 (80.0) | 0.9001 | 11/49 (22.5) | 3/8 (37.5) | 0.3592 |
| Cholesterol (%) | 56/238 (23.5) | 41/51 (80.4) | 5/5 (100.) | 0.2789 | 13/41 (31.7) | 3/5 (60.0) | 0.2098 |
|  |  |  |  |  |  |  |  |
